# Observations on the Case of Ann Fooks, in Reply to Dr. Yeats and the Rev. Mr. Grimshaw

**Published:** 1814-03

**Authors:** John Pulley

**Affiliations:** Bedford


*For the Medical and Physical Journal.*

### *Observations on the Case of Ann Fooks, in Reply to Dr. Yeats and the Rev. Mr. Grimshaw; by* Mr. John Pulley, of Bedford.

THE urinary aberrations of Ann Fooks have been a source of long discussion; and as they have afforded a stream still rolling upon us, a little time must yet be wanting to lead it to its ebb. Again, under matters as they go, I must exercise your patience, in vindication of truth, and the fair professional reputation of others and myself. The case of the afore-mentioned personage has originated a wide difference of opinion; such difference has produced discussion, and unavoidably of a controversial nature. The "complexion" of this controversy has been arraigned: it has been exhibited in dark colors; but I intreat any man *in his wisdom* to expound, under all the circumstances of the subject, how it could have differed. It certainly is much to be regretted that dispute has arisen; but, undoubtedly, it is not the fault of those who reject the reality of the case at issue. They sought not the controversy; they rested on their opinion, and would have remained in silence, had they not been goaded to the trial; but, having been compelled once to take the field, it will become their fault, and their stigma throughout life, if they shrink ignobly from the task imposed.

As a weariness may have arisen among some of your readers from this lengthened discussion, it may be matter of delight to them that a new character, in a double capacity, has presented himself to your notice. I allude to your medico-clerical correspondent for January. He has appeared before you as a physician and a minister of the gospel; he has given his medical opinion of the case in question, and offered his sentiments on the moral character of the woman. Had the Rev. J. S. Grimshaw, believing, as he must do, in the religious disposition of Ann Fooks, confined himself to the expression of such belief, the world would have seen him acting only according to the proper dictates of his holy office, and have applauded his intentions; but, unwilling to be circumscribed within the pale of his duty, he has become the champion pion of Dr. Yeats and the advocate of his opinion. He has deprecated personality, and severely rebuked attack on reputation; but I challenge him to adduce a more unjust, illiberal, or personal thrust at the fair fame of any man, than he has made at Mr. Gibbon. He has also, indirectly, given a fling at every medical person who has *presumed* to differ with him and the doctor.

But the most important point is yet to be noticed. Mr. Grimshaw has introduced, with great solemnity, a conversation which passed between himself and the lady of the theme, concerning the proposed employment of the catheter; and I am sincerely happy to have it in my power to corroborate what Mr. Gibbon has advanced on that part of the subject. Ann Fooks and her mother both acknowledged to me, in the presence of Mr. Gibbon, that the latter, when he mentioned the introduction of the instrument, offered to send for Dr. Yeats, or any other medical gentleman whom they might think proper to appoint. They told me also that they would not consent to its use, as Dr. Y. had asserted that the operation might be fatal to her. I well recollect in what way I replied: my expression was not very refined; I told Ann Fooks that the passage of the catheter would have done her no more harm than eating a piece of bread and cheese. Here let me state, that I do not for a moment accuse the doctor of the ignorance and folly which they ascribe to him by the supposed fatality likely to arise from the use of the catheter; but that such were the expressions, I most solemnly declare. And, after this, without noticing other circumstances, what credit is due to the assertions of these impostors? The observation, that Mr. Gibbon desired his patient to "make haste" to vomit, is too ridiculous and contemptible for refutation; and any man must libel his own understanding, to make it a subject of serious accusation.

Mr. Grimshaw has hinged much on the unimpeachable moral principles of Ann Fooks; but if it be proved that she has imposed upon Dr. Yeats in one way, it is not difficult to imagine that she may have imposed upon the reverend gentleman in another. Mr. Gibbon's statement has decidedly established the imposition as to urinous vomiting; and the *pious sister* of Ann Fooks, the immaculate and miraculous Ann Moore, of Tutbury, has evinced to the world that such a quality exists as religious hypocrisy, capable of eluding, for a long period of time, the detection of honorable and discerning men. Mr. Grimshaw ought to have confined himself to the expression of his sentiments on the moral and religious character of the woman. How different has been the conduct of Mr. Richmond and Mr. Martin! Barring two two or three objectionable words, they have not exceeded what may be considered as their duty; and in their performance of it they have been assisted by a third person, whom strangers, perhaps, will scarcely believe to be the identical Mr. Grimshaw who so unjustly and so profusely bespattered the character of Mr. Gibbon.

On reviewing the history of the diseases with which Ann Fooks is said to have been afflicted for twelve years, every one must be struck with the complication of her disorders and the heaviness of her sufferings; and it must be a matter of surprise to most persons that she has been able to sustain such unparalleled complaints. I have known her at least ten years, and am thoroughly satisfied that, at the period she was visited at Bedford by those medical gentlemen who signed the "written document," she was as fleshy in appearance as in the year 1804. We are invited to believe that but little, if any, difference exists between the terms "very thin" and "emaciation." Still, they carry a meaning of distinct import. A person may be *very thin*, yet possess the agility of a Vestris, and be able to wrestle with Herculean and Antæan vigour; but a state of *emaciation* implies *a loss* of flesh, which must arise from severe affliction or senescence. I allow that Ann Fooks may have been a weakly person for twelve years past; but I cannot admit that her afflictions have all along possessed such severity as related, as no human constitution could have resisted such formidable and continued maladies. She is reported to have labored for a great length of time under a "preying hectic," which, had it actually seized her frame, and maintained a long and obstinate abode, *must have shown itself by great emaciation.* On the 26th of June, 1812, not even the slightest tendency to emaciation existed. Her breasts appeared remarkably healthy: they were round and firm, and more prominent, as I observed in my former paper, than what is generally observed with women of her form. This state of the body was witnessed by Drs. Kerr and Alvey, and Messrs. Short and Gibbon, as well as by myself. Ten days subsequent to this period, Dr. Yeats asserts that her breasts were flaccid. In the interval, it does not appear that her general condition was at all altered. I must, therefore, leave it to the medical world to explain by what operation the flaccidity arose in ten days, which could not be effected by the most wonderful sufferings of ten years duration.*

The doctor has repeatedly used the term hectic, and, if

* Lucy Foster became much emaciated.

he he means to apply it to prove the inroads which were made upon her constitution in the former years of her illness, I have to answer, that no visible signs of greatly-impaired health were extant; but, if he intends by "hectic" merely to say that she was in a bad state of health, I admit the propriety of the term. I employed the same word, with the latter signification, when she was under my care at the Bedford Infirmary, because her symptoms were various, and I could not have given a concise designation of her state without omitting the mention of one or other of her complaints. But, during her residence at the Infirmary, she had no "preying hectic," nor was there the least appearance of her indisposition leading to dissolution. Her own report of her sufferings created suspicion in my mind long before her admission into the Infirmary. I recollect once visiting her, at her parish at Wootton, when she informed me that the previous day she had coughed up much more than a pint of blood.* About this time, I know that she had a slight spitting of blood, as I saw her expectorate some small portions mixed with mucus. But, at the period of the above visit, she had no symptoms of irritation, as must inevitably have attended a profuse sanguineous discharge from the lungs; *and on the day subsequent to such visit, she walked from her house at Wootton to Bedford, and returned home on foot, a distance of at least eight miles, without much time for resting, as I met her on the road both going and returning.* I know also, that from such time she was often accustomed to walk to Bedford, as I met her frequently.

An admirably precise account is given of the various turns of vomiting, with and without the aid of emetics; and it strikes me that this part is as dexterously planned by the woman as any portion of the fable. It gives a feature to the tale; and not a stone is left unturned to establish the validity of this marvellous case. We have adduced two or three instances of suppression of urine, combined with vomiting of blood; and because Ann Fooks is said to have endured the like symptoms, it seems to be hoped, although it is not expressed, that such coincidence may be insinuated as a lift to the probability of her story. She may have had suppression of urine and vomiting of blood, as well as the other instances. Let these circumstances be admitted; the doctor is welcome to the admission; they have their limit, and cannot avail him. They go not one step with him to prove the vomiting

* Dr. Yeats will recollect my mentioning this circumstance to him the day on which we visited Ann Fooks.

of of *pure urine*. Besides, it is not difficult to show why vomiting of blood should sometimes be connected with suppression of urine. The stomach is known to sympathize much and powerfully with all organic mischief, and in no instances more particularly than under affections of the urinary viscera. When suppression of urine arises from any cause, whether accompanied or unattended by secretion, vomiting, violent and frequent vomiting, will commonly supervene. Such repeated exertion must consequently induce a determination of blood to the part; and the ultimate result, where a laxity of vessels exists, may be a rupture or ulceration of one or more of them, and sanguineous effusion.

Still bearing on the case of contention, the doctor informs us that, on vomiting, the stomach did not altogether subside, but became hollow at its upper portion; and here he introduces a note, extracted from a paper by Sir Everard Home, contained in the Philosophical Transactions for 1808. Why is this quotation given? It must be to defend the possibility of vomiting *pure urine;* or it signifies nothing. But, if it be intended to prove this, it is as utterly unconnected with it as a bubble floating in the air. The remark of Sir E. Home applies to fluids admitted into the stomach through the medium of the mouth; Dr. Yeats's case allows only the consideration of fluids sent to that viscus by secretory or exhalent action: before, therefore, he can bind his quotation from the above paper to lend him any thing in argument, he must prove that the cardiac portion of the stomach only possesses a secretory or exhalent office. The doctor has again, with the same view, erroneously referred to the like quotation, at page 106 of your last Journal.

In another part we are acquainted that "the stomach never entirely subsided during the time of the suppression;" but that "it formed a reservoir for the reception of morbid secretions of its own disease, as well as of the urine." Yet Mr. Gibbon has positively stated that he examined the woman when no tumefaction existed; and at which time she had passed some days without vomiting urine. Be it admitted that the stomach is diseased; I am ready to allow it; but even were the doctor to prove the occasional residence of urine in that organ, I should be very slow to believe that it could at all times be found distended by that fluid or the "morbid secretions of its own disease." The existence of flatus, ever generated from a much impaired tone of the stomach, would more probably originate and continue a state of distention than one or the other; but even this could not always be operative, and Mr. Gibbon has proved by ocular ocular and tangible demonstration that the epigastric region was occasionally in a state of subsidence.

I have offered my theory concerning the vomiting of urinous particles with other matters, and although *facts** are of more "avail than any theory," let such theory be assailed by good argument, and by facts, well, thoroughly, and incontrovertibly established. The case of Ann Fooks will never shake it: it is founded on anatomy and the *known* laws of the animal economy; and whatever may have been the torpor of her glands, and the suspension or alteration of their secretions, so ingeniously hinted, and so exactly suited to the trying exigencies of the moment, she never could have vomited a quantity of pure urine. This liquid, *having once entered the circulation, and having become an intimately-blended part of the circulating fluid, must, at all times, have been disseminated through all parts of the system, and have been evacuated without the perfect character of urine.* Besides, it is confidently stated that the great distension of the stomach, and which never subsided, "was evidently owing to a collection of *great* morbid effusions into it." How was it then that such great morbid effusions could remain snugly and quietly in the stomach, exhaled, or secreted, like the urine, and as likely as the urine to give offence to their habitation, while the urine was freely and almost constantly discharged?

When Mr. Gibbon found that strong persuasive attempts could not influence the daughter and the mother to consent to the introduction of the catheter, he hinted a determination to expose their system of imposture. What was the consequence? In a short time she lapsed into a state of insensibility; for a few days she labored under stupor or delirium, and then, as if a talisman had dexterously touched her body and bid the waters flow, her urine returned to its right abode, and deigned to be expelled as nature has directed! The dream was at an end; stupor and delirium instantly suffered reason to assume her throne, and, thenceforward, the catheter was balked of its useful assistance at detection, and the residence of the food was relieved of the intrusion of an unnatural and unwelcome guest.

It appears that Elizabeth Triplow, and Lætitia Clarke, were the persons who played so conspicuous a part in the *honorable* and legal attestation of a falsehood. Like Ann Fooks, they imposed upon all around them. One swore positively that she personally witnessed vomiting of urine; the

* Has Dr. Yeats given us *the whole* of Dr. Girdlestone's observations on the Yarmouth case?

other, that she saw vomited a dark slimy matter, possessing a nauseous smell, like strong urine. One saw the crow actually take flight from the stomach; the other caught a glimpse of something as black! They took the oaths with great readiness, and with every appearance of truth; so says Dr. Yeats. But Mr. Short and Mr. Gibbon most distinctly affirm, that these women had previously informed them that they *never beheld any thing of the kind.* That these gentle and accommodating creatures swallowed the oaths with "great readiness, and with every appearance of truth," is not to be questioned. Indeed, I doubt not that they performed the ceremony as glibly and as readily as they would have accomplished the deglutition of an oyster. It is reported, that in London certain persons are found, on particular occasions, perambulating the precincts of our courts of justice with straw in their shoes—I advise the good Elizabeth and Lætitia to become companions of *the order of the straw*, and recommend their friends to propitiate their undertaking, and lead them to the purlieus of the Old Bailey.

An endeavour is still made to impress the minds of the public with the belief that the opinion formed on Ann Fooks's case by the medical gentlemen who differed from Dr. Yeats, was intended to apply to all instances of vomiting of urine. It is astonishing that the doctor can yet persist in this insinuation. I was one of the five medical characters opposed to him in the question, and he has acknowledged that I stated to him an opinion of urinous vomiting, similar to what I have described in my paper already published. Why, then, should he desire it to be understood that I could set my signature to a document, disclaiming, according to his erroneous judgment, even the idea of the discharge *of urinous particles* from the stomach?

With respect to Dr. Senter's case, it is argued that a stone might probably have existed in the bladder at one period, and have disappeared totally on examination after death. I dissent from this doctrine, especially in the case at issue. I do not believe that a calculous concretion, formed for a length of time, and distinctly tangible on repeated trials, could be so friable in its composition and all its parts, as to be easily crumbled and altogether evacuated with the urine. Nor is it probable, even if Lucy Foster vomited *pure urine*, that sabulous matter also should have been ejected from the stomach, as with her the urine often remained many hours in the bladder, and, consequently, much more than sufficient time was allowed for the formation and deposition of sabulous particles. It is observed in all cases where a strong tendency to the formation of calculous matter exists, that it rapidly rapidly takes place. It seems to arise almost instantaneously on the secretion of the urinary fluid, or the infundibula and pelves of the kidneys would not sometimes be found choked with it, in a concreted state; nor should we meet with those frequent instances of pain from the passage of a stone along the ureter. I have admitted that *urinous particles*, combined with other fluids, may be exhaled into the stomach and all other cavities of the body; but I have denied, and still deny, that urine, in its pure and unadulterated condition, can be so deposited.

Dr. Yeats asserts that "the component parts of urine *in solution* is urine," and that "nothing else can be made of it." Surely, the doctor is here quibbling in his reply to me, hoping to catch me trippingly in fault. I perfectly agree with his subtile definition of urine, as we find it flowing from its natural parts, subsequent to natural and healthy secretion; but, if he intend to tell me that *urinous particles*, when absorbed from the bladder, exhaled into the stomach, and evacuated at the mouth with other fluids, constitute vomiting of *pure urine*, or can be considered as urine, I deny the truth of his argument. Nothing can be pure in its nature and condition that is mixed with other substances. If a drunkard went into a tavern and called for a gill of brandy, would he give to it the name of the spirit if it smacked but faintly of his potent charm from being adulterated with other liquids? or, if I offered to the doctor a piece of money, composed of some base metal and a little gold, would he call it a guinea, and take it as the sterling representative of one pound *one*? I do not credit the report of Lucy Foster having vomited, in any way, an urinous fluid, unmixed with other matter; but, if she drank her urine, Dr. Y. does "not see how it was to be vomited unmixed with the contents of the stomach, taken in this way, any more than when it is passed into the stomach by the animal economy." Yet, in a former part of his paper, he has taken great pains to show how this could be effected, which will be readily perceived by referring to his note in page 21 of your Journal for January. In my prior communication, I purposely abstained from remarking on the discharge of urine from the navel, because Dr. Senter has not stated that he, or his medical friends, were evidences of the *fact*. We are told that no urinous fluid was discovered after death, as it was *all* discharged by vomiting and purging previous to dissolution. This may pass tolerably as an apology for the emptiness of the stomach and intestines; but how is it, that no taste or smell of urine existed in the several hydatids attached to the tubæ fallopianæ, and the enlarged ovaria, which contained a quantity tity of limpid fluid, and whence the liquids, *unfortunately*, could not escape; nor were they injured and disguised by fœtor, or by gangrene? I respect the name of Dr. Simmons, but I care not in what way he, or any other man, has estimated the "judicious remarks" of Dr. Senter. I repeat, in substance, what I have before stated, that the case is negligently drawn up, and that his observations at the conclusion of it are wanting both in science and judgment. I trust that this avowal will not, among liberal and candid minds, be thought improper. But I am accused of "blowing the breath of displeasure," and entertaining "disregard" for departed worth and excellence. I repel the charge with an honest conviction of its injustice; yet, at the same time, let it be distinctly understood that I am in the habit of thinking for myself, and shall not esteem it requisite to yield to the errors either of living or departed greatness. I may admire the genius, the erudition, and the style of Dr. Johnson; but it follows not that I am to subscribe to his failings, to become blind to his weak and puerile superstition. Men may be good and great; they may be amiable in disposition and excellent in understanding; yet still they are mortal, and liable to all the infirmities of their nature, consequently they must have a portion of error inseparable from their being. A generalissimo, though full of tactic, and hot to seek "the bubble reputation even in the cannon's mouth," may sometimes be found wanting at the hour of battle; an archbishop, though possessing much of the spirit of divinity, may not always be right in his scriptural annotations; and those great and good men, so extolled by Dr. Yeats, whose opinions, in general, are valuable, and whose characters are estimable, may have sometimes erred in their physiological and pathological remarks. Had I possessed the power, I have not the inclination, the folly, or the infamy, to attempt to raise myself by depreciating the reputation of other men: such conduct I disclaim, and I give to it my most unqualified abhorrence. But, though I revolt at such unmanly and inglorious proceeding, I will not yield to the presumption of the great fame of any man. I venture to hope that I know a little of my profession; and, surely, it is not too much to ask that I may be permitted to give an opinion. If I do this, it is done openly, it is before the public; and if I hazard, or have hazarded, a notion which cannot be maintained, let it be fairly canvassed, and candidly and manfully run down by the force of argument; but do not let great names, however worthy and exalted, be held up as bugbears to scare the reasoning faculty of man, and overwhelm the right of discussion. I repeat, that I have offered a theory on the vomiting of *urinous particles*, founded on anatomy and the *known* laws of the animal economy. Let it be refuted.

*Bedford, Feb.* 8, 1814. JOHN PULLEY.

