# Corrigenda

**Published:** 1814-04

**Authors:** 


### *CORRIGENDA.*

In our last, p. 203, lines 21 and 22, dele, "*nature and.*"

